# Phoenixin—A Pleiotropic Gut-Brain Peptide

**DOI:** 10.3390/ijms19061726

**Published:** 2018-06-11

**Authors:** Martha A. Schalla, Andreas Stengel

**Affiliations:** 1 Charité Center for Internal Medicine and Dermatology, Department for Psychosomatic Medicine, Charité-Universitätsmedizin Berlin, corporate member of Freie Universität Berlin, Humboldt-Universität zu Berlin, and Berlin Institute of Health, 12203 Berlin, Germany; martha.schalla@charite.de; 2 Department of Psychosomatic Medicine and Psychotherapy, Medical University Hospital Tübingen, 72076 Tübingen, Germany

**Keywords:** central nervous system, food intake, gastrointestinal tract, gut-brain axis, gut hormone, neuropeptide, orexigenic, phoenixin-14, phoenixin-20

## Abstract

Phoenixin is a recently discovered brain peptide initially thought to be restricted to reproductive functions. The subsequent identification of phoenixin’s expression in peripheral tissues was accompanied by the description of several other actions of this hormone, such as effects on behavior, sensory perception, memory retention, the cardiovascular system as well as food intake, pointing towards a pleiotropic role of this peptide. The present review will discuss the present knowledge on phoenixin and the signaling involved as well as highlight gaps in knowledge to stimulate further research.

## 1. Introduction

The bidirectional neuro-/humoral interaction between the central nervous system and the gastrointestinal tract—here, the enteric nervous system, gut hormones, and the gut microbiota are especially relevant—is involved in the regulation of several homeostatic functions [1]. In addition to gastrointestinal diseases such as irritable bowel syndrome [1], numerous other diseases such as obesity [2,3], psychiatric disorders like anxiety, depression [4], and autism spectrum disorder [5] as well as neurodegenerative diseases such as Parkinson’s disease [6] have been linked to dysfunctions of the so called gut-brain axis. The first studies reported that restoration of this axis e.g., by manipulation of the microbiota might improve the clinical course in patients [7]. Therefore, it is indispensable to understand the different elements of the gut-brain axis.

A crucial component of the gut-brain axis are neuropeptides signaling in both brain-gut and gut-brain directions [8]. Often initially discovered in the brain, several peptide transmitters are also—or even predominantly—expressed in the gastrointestinal tract such as nesfatin-1, peptide yy (PYY) and neuropeptide y (NPY) [8,9,10]. Recently, a novel peptide, phoenixin, was discovered in the rat brain and shown to exert several effects discussed in the present review. Since phoenixin was located in brain nuclei involved in the regulation of food intake such as the paraventricular nucleus (PVN), arcuate nucleus (ARC), ventromedial hypothalamus (VMH) and the nucleus of the solitary tract (NTS) [11] as well as in the gastrointestinal tract [12], a role in the brain-gut/gut-brain regulation of food intake was postulated and later confirmed by a study showing an orexigenic effect of phoenixin [13].

In light of the worldwide increasing rates of obesity and related diseases such as diabetes, cardiovascular diseases [14] as well as psychiatric comorbidities such as anxiety and depression [15], a deeper understanding of gut-brain signaling involved in the regulation of food intake along with pathophysiological alterations under conditions of obesity are necessary. The current review will present the state of knowledge on phoenixin expression, signaling, and effects as well as discuss its relevance in the context of the brain-gut axis.

## 2. Discovery of Phoenixin 

In 2013, the group around Samson developed a bioinformatic algorithm for the identification of unknown peptides [11]. On the basis of information from the Human Genome Project sequences encoding already identified peptides as well as gene-encoding receptors were excluded, while proteins containing a signal peptide or dibasic cleavage sites were further analyzed using the SMART database, SignalP database and BioRegEx database. Using this algorithm, the highly-conserved peptide, phoenixin, was discovered using NCBI BLAST (Basic Local Alignment Search Tool), an algorithm used for comparing newly discovered amino sequences with sequences of known proteins saved in the database of the National Center for Biotechnology Information [11]. Subsequently, phoenixin was detected in several species including humans, rodents, pigs, cows, chicken, xenopus, silurana, zebrafish and fugu in a highly-conserved manner with only slight differences in the amino acid sequence, e.g., one amino acid difference between human and rodent phoenixin and none between human and bovine phoenixin [11] giving rise to its physiological importance. Phoenixin was found in several amino acid lengths, namely 42, 36, 26, 20, 17, and 14 amino acid-containing peptides with phoenixin-20 and phoenixin-14 representing the predominant forms [11]. All peptides are derived from the same precursor sequence containing several dibasic residues and a C-terminal glycine as potential cleavage sites [11].

It remains unknown so far whether the different bioactive forms of phoenixin varying in length also differ in their bioactivity or bind to different receptors/receptor subtypes. It is important to note that regarding phoenixin’s effect on luteinizing hormone (LH) both, phoenixin-14 and -20, significantly augment the release; however, phoenixin-20 is effective at a lower concentration of 100 nmol, whereas 1000 nmol of phoenixin-14 are necessary to stimulate LH release in vitro [11].

## 3. Expression of Phoenixin

### 3.1. Central Expression 

First peptide level analyses indicated the highest concentration of phoenixin in the rat hypothalamus (2851 pg/g) followed by expression in peripheral tissues as described in detail below [11]. Detailed expression analysis using a self-generated phoenixin antibody targeting C-terminally-amidated phoenixin showed immunoreactive (ir) cells characterized by a diameter ranging from 10–15 μm in particular in the hypothalamus, namely in the magnocellular and parvocellular supraoptic nucleus (SON), dorsal hypothalamus, zona incerta, VMH, lateral hypothalamus, perifornical area, ARC, supraoptic retrochiasmatic nucleus and, to a lesser extent, in the PVN [11]. Double-labeling of the rat hypothalamus using phoenixin-14 and nesfatin-1 antibodies showed a high level of co-expression ranging from 70% to 86% in the ARC and PVN as well as in the lateral hypothalamic nucleus and VMH [16]. Phoenixin-14 ir cells were medium-sized and varying in shapes from round, multipolar, fusiform, elongated, polygonal, triangular, and piriform to droplet-shaped [16].

In addition to the hypothalamus, other areas expressed phoenixin immunoreactivity (IR), such as the substantia nigra, Edinger-Westphal nucleus, NTS, dorsal motor nucleus of the vagus, and area postrema with moderate intensity [11]. In addition, the median eminence, anterior and posterior pituitary showed phoenixin IR [11]. A subsequent study extended the knowledge on central expression sites and detected phoenixin-14 IR in the spinal cord superficial dorsal horn of cervical, thoracic, lumbar and sacral segments, especially in laminae I and II, but also in deeper laminae [17]. Positively labeled cells were found near the central canal and superficial layers of the spinal trigeminal tract partly extending to the medial and central nucleus of the solitary tract as well as to the nucleus ambiguous [17]. In sensory ganglia, namely the dorsal root, nodose, and trigeminal ganglion, phoenixin IR was detected in cells of a diameter between 25 and 40 μm, whereas no IR was found in the superior cervical ganglion sections [17]. A recent study detected a high density of phoenixin-14 IR in the central amygdaloid nucleus and the spinocerebellar tract of the medulla, while fewer positively labeled cells were found in the bed nucleus of the stria terminalis and raphe pallidus [12]. Interestingly, unlike the first study, no IR was found in the PVN [12]. Whether differently processed forms of phoenixin recognized by the antibodies used in these studies contribute to this controversial finding will have to be further assessed. Lastly, it is important to note that the recent study mostly detected phoenixin ir fibers, while only a few labeled cells were observed [12].

### 3.2. Peripheral Expression 

In addition to the expression in the brain, early on phoenixin was also detected in the periphery. The first study detected phoenixin by means of an enzyme-linked immunoassay (ELISA) predominantly in the heart (485 pg/g), thymus (307 pg/g), esophagus (298 pg/g) and stomach (274 pg/g) [11]. Furthermore, lower levels were found in the spleen (234 pg/g), pancreas (179 pg/g), lung (179 pg/g) and kidney (120 pg/g), while very low levels of phoenixin were detected in the gut, including the jejunum (102 pg/g), duodenum (36 pg/g), ileum (30 pg/g) and colon (2 pg/g) [11]. A recent immunohistological study detected phoenixin ir cells predominantly in crypts of the duodenum, jejunum and ileum as well as selectively in the outer endocrine islets of the pancreas, whereas other peripheral tissues showed no IR [12]. Another study examined skin tissues detecting bead-like structured phoenixin ir cell processes in the epidermis and dermis [18]. It is important to note that testis was investigated in several studies and consistently showed no phoenixin IR [11,12,19]; therefore, testis was subsequently used as a negative control [19].

A possible explanation for the partially-discrepant expression patterns of phoenixin IR could be different binding affinities of the used antibodies to different isoforms of phoenixin or related proteins such as MITRAC7 (mitochondrial translation regulation assembly intermediate of cytochrome c oxidase 7) which will be introduced in the next paragraph.

### 3.3. SMIM20/MITRAC7

As described above, phoenixin was discovered in the search of unknown highly conserved peptides on the basis of the Human Genome Project indicating that the nucleotide sequence encoding phoenixin in humans is located on chromosome 4 p15.2 registered under the name SMIM20—small integral membrane protein 20 [20]. Additionally, phoenixin-14 consists of an amino acid sequence appearing in the 7-kDa heavy protein structure of MITRAC7, an element of the MITRAC complex. The MITRAC complex consists of various COX1 (cyclooxygenase-1)-containing complexes, varying in their protein formation [21]. MITRAC7 is located in the inner mitochondrial membrane and able to interoperate with COX1 shortly after its synthesis. This interaction is dependent on the integration of nuclear-encoded subunits into MITRAC mediated by TIM21 (translocase of the inner mitochondrial membrane 21) [22]. Loss of MITRAC7 reduces COX1 activity around one-third through down-regulation of complex IV; conversely, overproduction of MITRAC7 leads to reduced activity of COX1 [22]. More precisely, lack of MITRAC7 results in COX1 accumulation, whereas overexpression of MITRAC7 impairs COX1 stability [22]. Therefore, MITRAC7 is indispensable for proper functioning of the respiratory chain and prostaglandin synthesis [22]. It remains to be investigated whether phoenixin interacts with complex IV or COX1 and has an impact on the energy status of the cell.

## 4. Mediation of Phoenixin’s Effects

Most of the functions of phoenixin including potentiation of gonadotropin-releasing hormone (GnRH)-dependent secretion of LH, augmentation of mRNA (messenger ribonucleic acid) expression in GnRH and kisspeptin neurons, stimulation or suppression of reproductive genes, e.g., *C/EBP* (CCAAT/enhancer-binding protein) mRNA [23], delaying the onset of estrus [24] and the increase of plasma vasopressin levels [25] were shown to be mediated through the GRP173 (G protein-coupled receptor 173). Conversely, blockade of the receptor using *GPR173* siRNA (silencing RNA) abolished these effects [23,24,25].

GPR173 (also called SREB3) has an amino acid similarity of 52 and 63%, respectively, with GPR27 and GPR85, and belongs to one of the orphan G-protein-coupled receptor subfamilies, namely the super conserved receptor expressed in brain (SREB) [26]. The name indicates the high conservation of the protein-coding mRNA region across vertebrate species giving rise to evolutionary importance [27].

GPR173 consists of seven transmembrane helical structures containing seven putative phosphorylation sites, mostly located in the third cytoplasmic loop [27]. Interestingly, phosphorylation often leads to desensitization and endocytosis of the receptor, accompanied by adaptor proteins such as β-arrestin, which is also involved in activation of the Erk (extracellular signal-regulated kinase) pathway [28] and cAMP (cyclic adenosine monophosphate) induction [29]. The third cytoplasmic loop additionally includes a lysine, a candidate for ubiquitination [27], leading to down-regulation by degradation of the protein, but also facilitating spreading of intracellular signals [30]. It is also important to note that GPR173 contains an asparagine residue in the N-terminal able to be N-glycosylated which simplifies recruiting ligands and interplays with the extracellular matrix [27]. Similar asparagine sites are found in the GnRH [31] and LH receptors [32] which are hereby essential for stability of the receptor and binding of the ligand. Both receptors are crucial components of the HPG (hypothalamic-pituitary-gonadal) axis and their similarity with the GRP173 underlines this receptor’s and phoenixin’s importance in reproduction and indicates possible resemblance in mediation and function of these receptors, a hypothesis to be further investigated.

GPR173 is widely distributed, especially in the brain, ovary and small intestine [26], co-localizing with the expression of GPR27 and GRP85 and highly expressed in areas with a high degree of plasticity such as the SON and PVN, hippocampal formation and olfactory system, suggesting implications in neural plasticity of the SREB family [33]. With increasing age *GPR173* mRNA expression is elevated in the ARC and medial preoptic area which can be negatively attuned by the administration of estradiol; in contrast, treatment with estradiol augments the GPR173 expression in young animals [34].

Detailed *GPR173* mRNA expression analyses in the rat brain indicated dense *GPR173* distribution reaching from the antroventricular periventricular nucleus, medial preoptic nucleus, PVN, SON and VMH to the dorsomedial hypothalamic nucleus [24]. Hippocampus, piriform cortex, lateral septum, bed nucleus of the stria terminalis, medial nucleus of the amygdala and the paraventricular nucleus of the thalamus expressed moderate to dense hybridization signals, while *GRP173* mRNA expression in the medial preoptic area, ARC, lateral hypothalamic area, and ventral premammillary nucleus was moderate [24].

Thus far, no endogenous agonists are known for this receptor group. Nonetheless, it has been postulated that GnRH-(1–5), a pentapeptide derived from GnRH after metabolization by endopeptidase (EP) 24.15, binds to GPR173 to inhibit the migration of cells, thereby prolonging wound healing and modulating neuronal migration during development in vitro [35,36]. GPR173 induces a canonical G protein-coupled receptor pathway. During this process GPR173 binds to β-arrestin 2-inducing phosphatase and tensin homologs resulting in mediation of GnRH-(1–5) effects [36]. In line with this finding, tumor cell surface membrane protein analysis indicated GPR173 in combination with neurotrophic tyrosine kinase 1 or ALK (anaplastic lymphoma kinase) to be a target for specific immunotherapy treating neuroblastoma [37]. Molecular docking studies showed that GPR173 is constitutively active and identified eight novel inverse agonists [38]. Improved memory recognition and anxiolytic effects induced by phoenixin can be abolished by cetrorelix, an antagonist of the GnRH receptor [36], indicating that these effects of phoenixin are mediated GnRH receptor-dependent.

Despite the fact that several studies give rise to a mediation of phoenixin’s effects via the GPR173 and binding of phoenixin to the GPR173 has been mentioned as unpublished observation [23], published studies investigating direct binding of phoenixin to the GPR173 (and also to the GnRH receptor) are still lacking. Nevertheless, the structurally similar GPR173 and GnRH receptor have been implicated in memory and reproduction similar to phoenixin. Interestingly, GnRH-(1–5) derived from GnRH exerts its effects via the GPR173, further corroborating the interaction between phoenixin, GPR173, and GnRH.

## 5. Effects of Phoenixin

### 5.1. Effects on Reproduction

The first biological action described for phoenixin was the potentiation of the GnRH-stimulated release of LH [11]. The stimulation of LH by phoenixin-14 and phoenixin-20 was shown to be GnRH-dependent with the GnRH receptor increasingly expressed after treatment with phoenixin [11]. These observations in cell cultures of the female pituitary were transferable to the HPG axis of female rats. Selective reduction of hypothalamic phoenixin levels using siRNA abolished the increase of GnRH receptor expression in the anterior pituitary [11]. Since this led to a retardation of estrus by about two days, a physiological role of phoenixin in this regulatory pathway can be assumed [11]. Another study determined the effect of phoenixin-14 injected intracerebroventricularly (icv) and showed an elevation of plasma GnRH after 5 min and a significant decrease below control levels after 30 min [39]. Moreover, phoenixin-20 injected icv increased plasma LH levels significantly at 5 and 15 min after injection in female rats [24]. Whether these prolonged actions are due to downstream signaling or reflect the half-life of phoenixin warrants further investigation. It is notable that phoenixin’s stimulating effect on the HPG axis was only detectable in female cells or animals [11,24], indicating that phoenixin is predominantly important in the female reproductive system. Subsequent studies should focus on other possible sex-dependent effects of this peptide.

**Figure 1:**
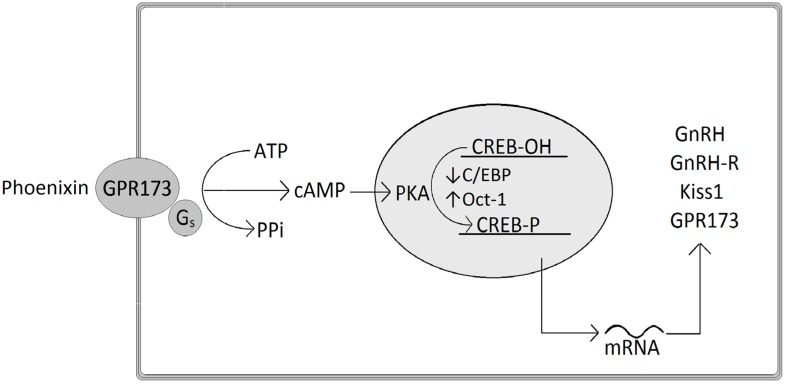
Phoenixin-20 stimulates the mRNA expression of GnRH, GnRH receptor, Kiss1 peptide, and GRP173 via the cAMP-PKA pathway and CREB phosphorylation mediated by the GPR173. Abbreviations: ATP, adenosine triphosphate; cAMP, cyclic adenosine monophosphate; C/EBP, CCAAT/enhancer-binding protein; CREB, cAMP response element binding protein; GnRH, gonadotropin-releasing hormone; GnRH-R, gonadotropin-releasing hormone receptor; GRP173, G protein-coupled receptor 173; Oct-1, octamer transcription factor-1; PKA, protein kinase A; PPi, Pyrophosphate.

At the molecular level, phoenixin-20 regulates the expression of GnRH by decreasing *C/EBP* mRNA and increasing Oct-1 (octamer transcription factor-1), both important transcription factors of the GnRH promoter (Figure 1). In detail, phoenixin stimulates cAMP levels and the phosphorylation of CREB (cAMP response element binding protein) and Erk 1/2, and acts by intracellular signaling of the cAMP-PKA (protein kinase A) pathway in order to induce a stimulation of *GnRH* mRNA expression [23]. Similarly, phoenixin-20 stimulates the expression of *kisspeptin* mRNA, a peptide also implicated in the release of GnRH at the onset of puberty [23]. These effects on intracellular cascades are assumed to be performed through the interaction of phoenixin with the orphan GPR173, since phoenixin-20’s in vitro effects on GnRH and kisspeptin neurons were suppressed following down-regulation of GPR173 resulting from the knockdown of the receptor using siRNA, since phoenixin has been shown to elevate the expression of GPR173 [23].

A study in patients with polycystic ovary syndrome (PCOS) described positive correlations between phoenixin-14 and LH, follicle-stimulating hormone (FSH), total testosterone, body mass index (BMI), and nesfatin-1 concentrations, and negative correlations with estradiol (E2) and fasting serum insulin [40]. Since these patients also displayed higher serum concentrations of phoenixin an implication of phoenixin-14 in the pathophysiology of PCOS has been suggested [40].

### 5.2. Effects on Food Intake 

Expression of phoenixin in hypothalamic nuclei involved in the regulation of food intake led to the hypothesis of a role of phoenixin in these homeostatic processes. Indeed, phoenixin-14 injected icv was shown to dose-dependently increase light phase food intake in rats [13]. Analysis of the underlying food intake microstructure using an automated monitoring device for the assessment of solid food intake in undisturbed rats indicated an increase in meal duration and meal size pointing towards a reduction of satiation [13]. It is to note that the orexigenic effect of phoenixin was delayed in onset and observed during the second hour post injection [13], likely associated with a recruitment of downstream mediators. This hypothesis warrants further investigation. This effect was also long-lasting and observed over the whole light phase as a stimulation of cumulative food intake. Interestingly, when injected during the dark phase phoenixin-14 injected icv did not (further) stimulate food intake in ad libitum fed rats [13], likely due to a stimulation of food intake by endogenous mediators already recruited during the dark phase [41,42].

Phoenixin-14’s effect on food intake seemed to be specific as no significant stimulation of water intake was observed [13]. However, food and water intake showed a significant correlation [13] pointing towards a secondary food-related increase of water intake. Moreover, no significant alteration of locomotor activity was detected [13] further corroborating phoenixin-14’s selective orexigenic effect. However, in mice phoenixin-14 and phoenixin-20 injected icv reduced body temperature [39], an effect that may secondarily affect food intake. This possible link should be further investigated.

The effect on food intake during the light phase was centrally mediated as the injection of similar doses intraperitoneally (ip) had no effect [13]. As mentioned above phoenixin IR was detected in several areas of the hypothalamus including the PVN, ARC, VMH and NTS [11]. Since the GPR173 is expressed in the SON and PVN, hippocampal formation and olfactory system [26], phoenixin might act locally in the brain to stimulate food intake. It remains to be investigated whether peripherally (gut or pancreatic) produced phoenixin can cross the blood-brain barrier or signal via the vagus nerve. Moreover, the role of gut- and pancreas-derived phoenixin such as an involvement in the regulation of immune functions, motility, or glucose control will have to be established.

Plasma analyses observed increased postprandial levels of phoenixin (50 pg/mL) in rats compared to levels before ingestion of standard diet (15 pg/mL) [19]. Interestingly, this increase was not observed in diet-induced obese (DIO) rats fed a high fat diet (HFD, 8–15 pg/mL), leading to a significant difference between pre-prandial plasma levels in normal weight and DIO rats [19], possibly indicating a desensitization of phoenixin signaling under conditions of DIO.

In humans, the effect on food intake remains to be investigated. An association of phoenixin with long term changes of body weight has been described with a positive association of phoenixin with BMI in patients with PCOS [40] or mild cognitive impairment [43], while in a cohort of male patients with different levels of anxiety this correlation was absent [44]. Therefore, this association should be further investigated to also identify possible confounding factors.

### 5.3. Effects on Sensory Perception

The observation of phoenixin IR in sensory and dorsal root ganglia after retrograde tracing of peripheral nerves using fluorogold [18] led to the examination of phoenixin’s effect on sensory perception. Phoenixin-14 injected intrathecally (it) did not alter pain perception during the tail flick test [17], whereby the tail of the mouse is warmed up and the movement of the tail indicates pain perception. However, in a model of visceral pain, the writhing test, ip injected phoenixin-14 reduced the number of constrictions and writhes in mice that received ip injections of acetic acid, indicating reduced visceral pain [17]. Moreover, phoenixin-14 injected subcutaneously (sc) provoked repetitive scratching by the hind paws, an effect abolished by usage of the kappa opioid receptor agonist, nalfurafine [18]. It has been proposed that phoenixin induces sensations of itch after secretion from primary afferents possibly via release of dynorphin which inhibits spinal inhibitory glycine/gamma-aminobutyric acid neurons in a kappa opioid-dependent fashion leading to a disinhibition of spinal neurons [18].

### 5.4. Effects on Behavior and Anxiety

Phoenixin-14 and phoenixin-20 were both shown to induce anxiolytic effects. Mice injected icv with phoenixin-14 or phoenixin-20 at a dose of 25 nmol displayed less anxiety and increased explorative behavior in the open field and elevated plus maze tests indicated by increased time spent in the illuminated center of the open field box and in the open arms instead of the closed arms of the elevated plus maze 15 min after injection [39]. It is important to note that (besides icv application) microinjections of phoenixin-14 directly into the anterior hypothalamic area also led to anxiolytic behavior, whereas microinjections into the amygdala did not [39], giving rise to the site of action. Whether the amygdala is indirectly/further downstream involved in phoenixin’s anxiolytic actions warrants further investigation. The GnRH receptor antagonist, cetrorelix was able to inhibit the anxiolytic action of phoenixin-14 and phoenixin-20 [39]. Whether this reflects a direct interaction of phoenixin with the GnRH receptor or is due to a downstream activation of the receptor will have to be further investigated. The effect on anxiety seemed to be specific as other behaviors such as locomotion were not significantly altered following icv injections in mice [45] or rats [13].

In humans, one study described a negative association of phoenixin plasma levels with patient-reported anxiety levels (assessed using the GAD-7, Generalized Anxiety Disorder 7 questionnaire) in a cohort of obese men [44]. Moreover, using a median split for GAD-7 levels the group with lower anxiety displayed 25% higher phoenixin plasma levels compared to the moderate anxiety group with higher GAD-7 levels without reaching significance (*p* = 0.068) [44] leading to the hypothesis that phoenixin might be involved in the reduced experience of anxiety in these patients. This should be further assessed in a longitudinal study design. The association of phoenixin with anxiety is likely to be specific as no correlations were observed between phoenixin and reported depressiveness or perceived stress [44].

### 5.5. Effects on Memory 

Mice undergoing object and location recognition memory tests displayed enhanced and prolonged memory when treated icv with phoenixin-14 [45]. Direct bilateral injections into the hippocampus enabled the rodents to memorize objects and spatial details even after short training periods of 5 sec, whereas vehicle-treated animals failed [45]. Additionally, three days after the end of the training phoenixin-14-treated animals presented good memory retention, whereas control animals showed worse memory [45]. The effect of phoenixin-14 was completely abolished by pretreatment with the GnRH receptor antagonist, cetrorelix [45], indicating that phoenixin-14’s impact on memory retention is mediated via direct or downstream GnRH receptor signaling. Lastly, phoenixin-14 was shown to abolish the memory impairment induced by amyloid-β_1–42_ and scopolamine [45], highlighting phoenixin-14 as a potential drug target in the treatment of e.g., Alzheimer’s disease, a disease associated with short-term memory impairment and loss of attention control, reasoning, orientation and language affecting the activities of daily living [46].

However, clinical studies did not show differences in phoenixin-14 levels comparing patients with mild Alzheimer’s disease, mild cognitive impairment and subjective memory complaint [43]. Whether peripheral concentrations do reflect changes in the brain will have to be further investigated. It is to note that in patients with subjective memory complaints phoenixin levels positively correlated with immediate word list recall tested using the Rey Auditory Verbal Learning Word List test (RAVLT), whereby the participant reads a list of 15 words and then has to repeat all the words of the list he/she can remember immediately afterwards [43] indicating that low plasma level of phoenixin could be a predictive parameter in prodromal stages of diseases accompanied by memory complaints. On the contrary, in patients with mild cognitive impairment plasma phoenixin levels correlated negatively with logical memory [43] supporting the idea that phoenixin contrarily is affected by/affects immediate recall and logical memory.

### 5.6. Effects on the Cardiovascular System

Since phoenixin-14 was detected at fairly high concentrations of 550 pg/g in the heart tissue using ELISA [11], an involvement in cardiovascular functions has been hypothesized. In a model of cardiac ischemia using the Langendorff technique, whereby isolated hearts are brought into an ischemic state for 30 min followed by 120 min of reperfusion, cardiac phoenixin protein levels were elevated after reperfusion [19], possibly pointing towards cardioprotective effects of the peptide. Interestingly, in DIO rats fed a HFD this elevation was not observed [19], possibly associated with obesity-related cardiovascular malfunction, a hypothesis to be further investigated.

Under basal conditions, perfusion of isolated rat hearts with phoenixin-14 decreased myocardial contractility and relaxation as indicated by reduced left ventricular pressure (LVP) [19]. This decrease was associated with an enhanced phosphorylation of protein kinase B, eNOS (endothelial nitric oxide synthase) and Erk 1/2 [19] giving rise to the intracellular pathways involved. It is notable that after ischemia, reperfusion with a solution containing phoenixin-14 improved recovery of LVP and normalized left ventricular end diastolic pressure (LVEDP) at the end of the reperfusion [19]. Phoenixin-14 also reduced the infarct size after ischemia, expressed as the percentage of left ventricular mass, from 73% in hearts undergoing ischemia and perfusion without phoenixin to 34% with perfusion of phoenixin for 20 min at the beginning of the perfusion [19]. Hereby, increased phosphorylation levels of all reperfusion injury salvage kinase (RISK) elements and STAT3 (signal transducer and activator of transcription 3) from the survivor-activating factor enhancement (SAFE) cascade may be involved along with a phoenixin-14-induced decrease of pro-apoptotic factors such as Bax (Bcl-2-associated X), Caspase 3, Cytochrome C, and p38 and an increase of the anti-apoptotic protein Bcl-2 (B-cell lymphoma 2) [19]. These effects were attenuated by specific inhibitors of intracellular pathways such as wortmannin (PI3K-inhibitor), L-NIO (NOS-inhibitor), PD098059 (MAPKK1-inhibitor) and 5-hydroxydecanoate (mitochondrial ATP-sensitive K^+^ channel inhibitor), all necessary for cardioprotection [19]. Interestingly, similar effects of phoenixin-14 during reperfusion with phoenixin after ischemia were detected in hearts isolated from DIO rats fed a HFD [19]. Whether this cardioprotective effect also occurs in vivo in DIO rats will have to be further investigated since cardiac phoenixin levels did not rise after reperfusion in these rats. These positive effects of perfusion with phoenixin also on hearts of obese organisms at low levels (100 pmol/L) [19] led to the speculation of phoenixin being a possible drug candidate for post-ischemic conditions.

Since phoenixin was also detected in the SON [11], phoenixin-20’s effect on vasopressin and oxytocin expression was examined. Icv injection of phoenixin-20 resulted in an increase of circulating vasopressin levels 20–30 min after injection [25]. Blockade of the GPR173 using *GPR173* siRNA administered into the brain ventricle for two consecutive days completely abolished this effect [25]. In vitro, phoenixin-20 depolarized magnocellular neurons followed by augmented action potential frequency leading to stimulation of vasopressin hormone expression in hypothalamic explants as well as to gene activation in the SON. In contrast, no alterations in oxytocin protein expression in vitro, or plasma levels in vivo were induced by phoenixin-20 [25]. It remains to be investigated whether these alterations in vasopressin levels have effects on fluid homeostasis and/or vascular parameters.

## 6. Conclusions

**Figure 2:**
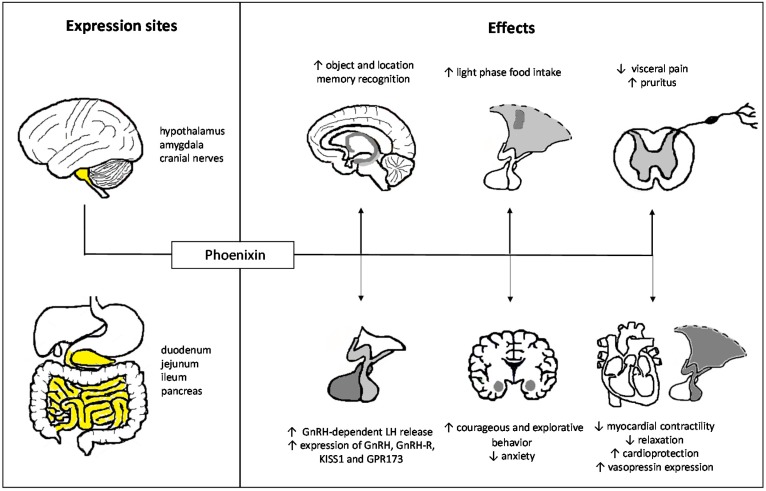
Pleiotropic effects of phoenixin. ↑, increase; ↓, decrease. Highlighted areas in yellow represent expression sites of phoenixin.

The past years have witnessed a great increase in our knowledge on the effects of phoenixin which extend far beyond the initially described role in reproduction highlighting rather a pleiotropic role for this peptide with effects also on food intake, perception, anxiety, memory, and cardiovascular functions (Figure 2). Since phoenixin is expressed both in the brain and the gut a communication via the gut-brain axis can be assumed. Despite the fact that the GPR173 is yet to be de-orphanized, mounting evidence points towards a mediation of phoenixin’s effects via this receptor. Further studies also using knock-in/knock-out models will help to further unravel the physiological actions of phoenixin with a particular focus on the role of peripheral (gut- and pancreas-derived) phoenixin.

## Abbreviations

PYY	Peptide yy
NPY	Neuropeptide y
PVN	Paraventricular nucleus
ARC	Arcuate nucleus
VMH	Ventromedial hypothalamus
NTS	Nucleus of the solitary tract
LH	Luteinizing hormone
SON	Supraoptic nucleus
ir	Immunoreactive
IR	Immunoreactivity
ELISA	Enzyme-linked immunosorbent assay
MITRAC7	Mitochondrial translation regulation assembly intermediates of cytochrome c oxidase
SMIM20	Small integral membrane protein 20
COX1	Cyclooxygenase-1
TIM21	Translocase of the inner mitochondrial membrane 21
GnRH	Gonadotropin-releasing hormone
mRNA	Messenger ribonucleic acid
C/EBP	Ccaat/enhancer-binding protein
GPR173	G protein-coupled receptor 173
siRNA	Silencing RNA
SREB	Super conserved receptor expressed in brain
Erk	Extracellular signal-regulated kinase
cAMP	Cyclic adenosine monophosphate
HPG axis	Hypothalamic-pituitary-gonadal axis
EP	Endopeptidase
ALK	Anaplastic lymphoma kinase
Icv	Intracerebroventricular
Oct-1	Octamer transcription factor-1
CREB	cAMP response element binding protein
PKA	Protein kinase A
PCOS	Polycystic ovary syndrome
FSH	Follicle-stimulating hormone
BMI	Body mass index
E2	Estradiol
ip	Intraperitoneal
DIO	Diet-induced obesity
HFD	High fat diet
it	Intrathecal
sc	Subcutaneous
GAD-7	Generalized Anxiety Disorder 7 questionnaire
RAVLT	Rey Auditory Verbal Learning Word List test
LVP	Left ventricular pressure
eNOS	Endothelial nitric oxide synthases
LVEDP	Left ventricular end diastolic pressure
RISK	Reperfusion injury salvage kinase
STAT3	Signal transducer and activator of transcription 3
SAFE	Survivor activating factor enhancement
Bax	Bcl-2-associated X
Bcl-2	B-cell lymphoma 2
PI3K	Phosphoinositide-3-kinase
L-NIO	N(5)-(1-Iminoethyl)-L-ornithine
MAPKK	Mitogen-activated protein kinase kinase
ATP	Adenosine triphosphate
